# Harnessing the B Cell Response in Kidney Transplantation – Current State and Future Directions

**DOI:** 10.3389/fimmu.2022.903068

**Published:** 2022-06-09

**Authors:** Imran J. Anwar, Isabel F. DeLaura, Qimeng Gao, Joseph Ladowski, Annette M. Jackson, Jean Kwun, Stuart J. Knechtle

**Affiliations:** Duke Transplant Center, Department of Surgery, Duke University School of Medicine, Durham, NC, United States

**Keywords:** B cell, antibody-mediated rejection, clinical trial, sensitization, kidney transplantation, immunosuppression

## Abstract

Despite dramatic improvement in kidney transplantation outcomes over the last decades due to advent of modern immunosuppressive agents, long-term outcomes remain poor. Antibody-mediated rejection (ABMR), a B cell driven process, accounts for the majority of chronic graft failures. There are currently no FDA-approved regimens for ABMR; however, several clinical trials are currently on-going. In this review, we present current mechanisms of B cell response in kidney transplantation, the clinical impact of sensitization and ABMR, the B cell response under current immunosuppressive regimens, and ongoing clinical trials for ABMR and desensitization treatment.

## Introduction

Although the role of alloantibody in kidney transplant rejection has long been acknowledged (1), efforts to elucidate and target mechanisms of rejections have focused primarily on T cell as opposed to B cell responses, partially due to the obvious role of cellular immunity in early graft rejection (2, 3). T cell-centric research in transplantation has produced immunosuppressive regimens that successfully target T cell activation and proliferation, dramatically improving short-term transplantation outcomes. Subsequently, rates and severity of acute and T cell-mediated rejection (TCMR) have decreased over the last 5-6 decades. Currently, 1-year survival rates following kidney transplantation are at an all-time high of 98.11% for living donor and 94.88% for deceased donor transplants, based on the 2020 Scientific Registry of Transplant Recipients (SRTR) report (4). However, long-term graft survival has not seen such dramatic improvements (5). Unsurprisingly, transplantation elicits both T and B cell immune responses, and while TCMR can be usually successfully treated, indolent antibody-mediated rejection (ABMR) has become the dominant mode of allograft injury and contributor to decreased survival (6, 7). Calcineurin inhibitors (CNIs), steroid treatments, and T cell depletion reliably reverse TCMR; however, these therapies are not effective in reversing ABMR. Therefore, a mechanistic understanding of the B cell immune response to transplantation is necessary to develop effective therapeutics and prolong graft survival.

Post-transplant B cell immune responses involve several unique populations. The downstream effector cells, plasma cells (PC), play a key role in producing immunoglobulin products (8, 9). They may be generated following germinal center (GC) formation, or extrafollicularly from memory B cells following an anamnestic response (10). Given the complexity of B cell response, treatment of ABMR will likely require targeting of multiple processes, such as B cell activation and proliferation, plasma cell differentiation, antibody production, and complement activation, in contrast to the singular target of TCMR treatments: the T cell. In this review, we present current mechanisms of B cell response in kidney transplantation, the clinical impact of sensitization and ABMR, B cell response under current immunosuppressive regimens (**Table 1**), and ongoing ABMR and desensitization treatment clinical trials.

**Table 1:** B cell response under current immunosuppressive regimens.

Agents		Mechanism of Action	B cell depletion	B cell differentiation	GC response	T_Fh_ differentiation	Plasma cell depletion	References
**Induction**								
	rATG	Anti-thymocyte globulin		x		x	?	71, 73, 74, 79
	Alemtuzumab	Humanized anti-CD52 mAb	x			?		80–82
	Basilixumab	Anti-IL-2R⍺ mAb				?		87–88
	Rituximab	Anti-CD20 mAb	x			?		83–85
**Maintenance**								
	CNI	Calcineurin inhibitor		x		x		90–94
	MMF, MPA	Inhibitor of guanine nucleotide synthesis		x				93, 95
	Steroids	Multiple, inhibition of NF-ĸB				?		98, 99
	Rapamycin	mTOR inhibitor			x	x		96, 104–109
	Belatacept	CTLA-4-Ig, CD80/86-CD28 blockade			x	x		110–114

## Physiology of the B Cell Response

B cell development and maturation has been expertly reviewed elsewhere; thus, we aim to briefly review the lineage and critical steps in B cells maturation as they pertain to allotransplantation and the resultant T cell-dependent antigen activation (43–45). B cell development can be divided into three periods: B cell receptor (BCR) recombination, B cell activation and affinity maturation, and terminal differentiation.

The birth of a B cell occurs in the primary lymphoid tissue (either bone marrow or fetal liver) with the development of a pre-pro-B cell from a common lymphoid progenitor cell. The bone marrow serves as the site of BCR development. A series of functional rearrangements of the heavy chain V, D, J and light chain V and J genes, supported by the survival factor IL-7 which is produced by the surrounding stromal cells, results in a BCR with reactivity to a broad array of foreign antigens (46). BCRs that are autoreactive are negatively selected against through either receptor editing of the Ig light chain or cellular apoptosis. The surviving immature B cells are able to exit the bone marrow as transitional B cells to complete maturation in the spleen (47).

The mature naïve B cell then circulates through peripheral blood and lymph channels in search of a cognate antigen (48). In the context of transplantation, this is most likely alloantigen derived from donor human leukocyte antigen (HLA). The BCR/antigen interaction commonly occurs in the secondary lymphoid tissues (spleen, lymph nodes, and Peyer’s patches) due to the persistent homing of B cells to these tissues. A critical component of this homing and migration is the chemokine CXCL13 which is produced by the T follicular helper (Tfh), follicular dendritic, and stromal cells and serves as the ligand for CXCR5. Specialized antigen presentation cells (APCs) within lymphoid follicles display antigen to the BCR.

A B cell is triggered to proliferate in response to a sufficiently strong co-receptor (e.g. toll-like receptor) stimulation or highly multivalent antigen that successfully activate a BCR signal. These responses are considered T-independent. The surrounding APCs and stromal tissue produce survival factors such as the TNF cytokines, B cell activating factor (BAFF), and a proliferation-inducing ligand (APRIL), which are thought to be essential to B cell survival in T-independent B cell proliferation (49).

B cells that respond to a low valency antigen will commonly progress down a T-dependent pathway and receive secondary signaling and cytokine stimulation from CD4^+^ Tfh cells. Following initiation of the T-dependent response, the B cell ultimately becomes one of the following three: short-lived plasma cells, memory B cells, or GC B cells. As expected, there are a significant number of cytokines involved in the B cell differentiation and proliferation pathway that are expertly reviewed elsewhere (50). A few cytokines noteworthy to this discussion are IL-4, IL-10, and IL-21— cytokines responsible for promoting B cell proliferation, class switch recombination, and differentiation. The factors associated with the B cell fate are under investigation; antigen affinity, BCR isotype, and exposure time to antigen may play a role (51–53). Given the role of long-lived antibody production in transplantation, GC B cells are of particular relevance to this review.

The GC is histologically organized into light and dark zones containing B cells in differing stages of cell division. Within the GC, B cells proliferate in large part due to the B-Tfh cell interaction consisting of multiple surface protein interactions and Tfh secreted cytokines. Following requisite activation, B cells undergo proliferation and somatic hypermutation, the process by which the variable region of the BCR is altered to produce Ig of varying affinity. Ideally a balance is struck in which the BCRs with the highest affinity for antigen, but not reactive to self-antigen, are selected. Disruption of this balance can lead to the development of auto-immunity. Selected GC cells will ultimately undergo class switch recombination from IgM to IgG, IgA, or Ig. Proteins important to activation and differentiation include the B cell class II HLA complex, CD40, ICOS ligand, and CD80/86 and the T cell TCR, CD40L, ICOS, and CD28 (54, 55). From a cellular standpoint, there is also growing evidence to suggest that certain subsets of Tfh cells also predispose the B cell to IgG and IgM production, the isotypes associated with complement fixation (56–59). Research into the role of these Tfh subsets in the context of allotransplantation is underway. Finally, as mentioned previously, IL-21 is a cytokine produced by Tfh cells with significant importance in B cell development, differentiation, and function.

IL-21 is thought to be critical to generation and maintenance of long-lived plasma cells and the differentiation of B cells into plasma cells capable of producing the complement-fixing IgG_1_, IgG_3_, and IgM (50), and potentially leads to long-lived plasma cell development through activation of the STAT3 signaling pathway (60–62). B cells that undergo this process terminally differentiate into either memory B cells or long-lived plasma cells. These cell populations are of importance to transplant recipients as their development results in a cellular reservoir capable of producing donor-specific antibody (DSA) for years after exposure. It is unclear how these cells survive for years; however there is likely a combination of external (cellular and molecular niches) and internal (anti-apoptosis and differentiation factors) influences (63).

Robust B cell response following transplantation has been consistently reported and is particularly relevant in the discussion of sensitized recipients or those experiencing ABMR. Below, we address the role of B cell response in these populations.

## HLA Sensitization

Sensitized recipients are those previously exposed to foreign HLA from pregnancy, transfusion, or prior transplant, resulting in the formation of HLA class I and class II antibodies. The role of these alloantibodies in transplant rejection was initially characterized in Patel and Terasaki’s landmark study, which defined positive crossmatch as the lysing of donor cells when mixed with recipient serum, and found a correlation between positive crossmatch and early graft loss (1). In the current era, HLA antibodies are detected in patient sera utilizing large HLA coated bead panels, providing greater sensitivity and specificity than earlier cell panels (64). Detection of donor specific HLA antibodies (DSA) in recipient sera is associated with increased ABMR risk and reduced allograft survival (65, 66).

HLA sensitization induced by previous transplants appears consistently as the strongest sensitizing event, while transfusion is associated with limited HLA antibody production in strength and breadth (67, 68). Pregnancy induced sensitization correlates with the number of live births, the HLA mismatch, and homozygosity in the mother’s HLA phenotype (69). A study examining the impact of sensitization route on long-term allograft survival revealed that highly sensitized recipients sensitized *via* previous transplants lost grafts at higher rates than those sensitized *via* pregnancy or transfusion (68).

New assays to detect HLA-specific B cell memory cells are advancing our understanding of B cell memory generation in transplant candidates and the potential ABMR risk that has historically been undetected (70, 71). Pregnancy and previous transplants have been shown to elicit HLA specific B memory cell generation, which may outlast serum HLA antibodies and pose an increased risk for post-transplant anamnestic alloantibody responses even when DSA is absent at time of transplantation (70, 72).

Historically, broad HLA sensitization has limited a candidate’s likelihood of finding a compatible donor resulting in prolonged waiting times. New organ allocation schemas in the United States now assign increasing allocation points according to the breadth of the patient’s sensitization (calculated panel reactive antibody, cPRA) and mandate national organ sharing for 100% cPRA candidates, thereby prioritizing the matching of compatible allografts to sensitized patients (73). Furthermore, the new allocation system with a larger donor pool has provided opportunity to transplant some of these highly sensitized candidates across low level DSA or DSA specific to HLA antigens that are expressed at lower levels on cells (HLA-C and -DP) are associated with lower risks of ABMR and limited impact on allograft survival.

Yet despite this prioritization, transplant inequity still remains for the most highly sensitized candidates (cPRA ≥ 99.9%) due to competition for kidney donors with rare or homozygous HLA antigen phenotypes (74, 75). The Eurotransplant Acceptable Mismatch allocation program for highly sensitized candidates is based on selecting donors whose HLA phenotype includes HLA antigens of the recipient’s phenotype as well as acceptable HLA antigens to which the recipient has never been sensitized. Reports by Heidt et al. (76, 77) show that highly sensitized recipients transplanted within this program experience lower rejection rates and allograft survival equal to non-sensitized patients.

## Antibody-Mediated Rejection

Antibody-mediated rejection (ABMR), a clinicopathological diagnosis that was first formally defined in 2003 (78), has gained importance in kidney transplantation and is now thought to be the primary driver of late graft failure. In their 2012 landmark study, Sellarés et al. (6) showed that ABMR was responsible for the majority of late graft failures. Several studies have since confirmed the impact of ABMR on short- and long-term outcomes. A meta-analysis demonstrated rates of acute ABMR (less than 1 year) between 3-12% and chronic ABMR between 5-20% following kidney transplantation (79), with rates up to 50% following positive cross-match living donor kidney transplant (80).

The definition and impact of ABMR has substantially evolved since the establishment of the Banff Criteria in 2003, allowing for improved diagnostic sensitivity and predictive value of graft outcome. The 2017 Banff criteria now incorporate C4d deposition and ABMR-related gene transcripts as a surrogate for DSA (81). It is now accepted that ABMR represents a wide spectrum of lesions at different time points following antibody-mediated injuries, ultimately leading to a spectrum of clinical phenotypes (82). Importantly, biopsies classified as no rejection displayed evidence of increased ABMR-related transcripts in patients with elevated DSA titers, highlighting the high prevalence of ABMR and likely under-diagnosis with current diagnostic modalities (83).

In recent years, the relationship between DSA and ABMR has become more clearly defined, and detection and precise characterization and quantification of DSA against HLA have become SOC following widespread use of Luminex-based assays. Lefaucheur et al. (84) convincingly show that patients who undergo transplantation with pre-existing HLA-DSA fare worse compared to patients without HLA-DSA, and that rates of ABMR are directly correlated to pre-transplant levels of HLA-DSA. However, despite higher rates of ABMR in patients with pre-existing DSA, ABMR following *de novo* DSA production portends to worse outcomes with a more aggressive molecular phenotype (85). Importantly, patients with ABMR in the absence of HLA-DSA have improved survival compared to HLA-DSA positive patients (86) and a distinct transcriptional signature (87).

### Contemporary Challenges With ABMR

Diagnosis and treatment of ABMR remains challenging. Subclinical rejection, the presence of rejection in kidney recipients who have neither clinical nor laboratory evidence of rejection, is common with incidence up to 30% in the first 3 months post-transplant (88). Subclinical ABMR portends to poor long-term graft survival; subclinical ABMR at 1-year is independently associated with a 3.5-fold increase in graft loss (89).

Given the invasive and expensive nature of kidney biopsies, several non-invasive alternatives have been pursued in the hopes of accurately diagnosing rejection and ABMR in particular. Donor-derived cell-free DNA, blood-based molecular biomarkers, urinary biomarkers, and tissue molecular diagnostics all constitute promising venues and have been reviewed by Westphal and Manoon (90).

Additionally, it is well described that interpretation of renal allograft biopsy pathology is limited by inter-observer variation and poor inter-observer agreement (91). Advances in machine learning and artificial intelligence have led to the emergence of digital pathology, paving the way for more accurate and reproducible interpretation of renal pathologies (92, 93). The Banff Digital Pathology Working Group (DPWG) was founded in 2019 at the joint Banff/ASHI meeting to promote and support the development and integration of digital pathology into clinical practice and research protocols (94).

Despite the prevalence of ABMR in kidney transplantation, there are currently no FDA-approved treatments for ABMR (95). In part, ABMR treatment has not been optimized due to the constantly evolving definition of ABMR and our incomplete understanding on the relationship between ABMR and DSA. Despite this, several different therapies have been evaluated in the past and have been extensively reviewed elsewhere (96–99). However, most studies of ABMR treatment are confounded by a heterogenous patient population, small sample size, and varying definition and chronicity of ABMR (96, 97). Intravenous Immunoglobulin (IVIg) and plasmapheresis are often considered standard of care (SOC) despite lack of evidence of efficacy or safety (79). Importantly, the current FDA-approved primary endpoints, 1-year graft and patient survivals, are not appropriate given excellent clinical outcomes under modern immunosuppression (>95% survival at one year) (81). Therefore, it is essential to establish validated surrogate endpoints is essential to develop and trial novel ABMR treatments (100).

## B Cell Response With Current Immunosuppression

### Induction Therapy

Greater than 90% of all kidney transplants include induction agents as part of their immunosuppressive regimen in the United States, with 60% receiving lymphocytes-depleting agents (usually in the form of rabbit Anti-Thymocyte Globulin (rATG)) and 20% receiving non-depletional agents (IL-2 receptor antagonist, Basilixumab) (4, 101). Alemtuzumab, a humanized anti-CD52 mAb, is used in several transplant centers despite lack of FDA approval for use in solid-organ transplantation. Induction agents have been shown to effectively prevent occurrence of early acute rejection and are now standard of care (102).

Rabbit Anti-thymocyte globulin (rATG) is made by immunizing rabbits with human pediatric thymi and purifying the IgG fraction, resulting in a polyclonal agent that bind multiple epitopes on T cells, causing both complement-dependent lysis and apoptosis and sustained T cell depletion (11). However, the effect of rATG on B cell response is less clearly understood and is thought to occur through several distinct mechanisms. First, the human thymus contains a small percentage of B and plasma-cells (103) and rATG contains antibodies against known B and plasma cell markers (11). Zand et al. (12) showed that rATG triggers B cell apoptosis *in vitro*, confirming that rATG exerts a direct effect on B cells. However, B cell depletion is typically minimal following rATG induction in kidney transplantation, perhaps due to the low dose commonly used (1.5mg/kg/day for 4 to 7 days) (13, 104–107). Second, rATG inhibits B cell differentiation and promotes B cell proliferation *in vivo* (14). Despite no significant B cell depletion, rATG induction in humans reduces frequency of memory B cells and class-switched B cells, likely secondary to impaired B cell differentiation (13). Finally, given that rATG causes pan-T cell depletion, including depletion of T follicular helper cells, T-cell-dependent activation of allo-reactive B cells is likely disrupted following rATG induction.

Alemtuzumab, a humanized anti-CD52 mAb, causes profound B cell, T cell, NK cell, and monocyte depletion and is thus used in select centers as an induction agent. B cell depletion is immediate and results in suppressed B cell population for 6 months, followed by repopulation that achieve absolute counts exceeding that of pre-transplantation levels (15–17). Naïve B cells are preferentially depleted (suggesting partial resistance of memory B cells to alemtuzumab), followed by repopulation driven by transitional and pregerminal center B cells that promote long-term expansion and dominance of naïve B cells. Furthermore, B-regulatory cells (BRegs) are increased (16). Todeshini et al. (17) reported that alemzutumab induction was associated with DSA production and worse long-term outcomes, suggesting the B-cell depletion following alemtuzumab induction may promote chronic humoral response against the allograft.

Rituximab, a mouse/human chimeric monoclonal antibody against CD20, potently depletes B cells and is often use in the context of highly sensitized kidney transplantation or ABO incompatible kidney transplants, to prevent antibody-mediated rejection given their higher immunologic risk profile (20). Sustained peripheral depletion (<5 CD19+ cells/µL) is readily achieved after a single dose (375 mg/m^2^ BSA) in less than 3 days and last for up to 12-14 months (21). The repopulated B-cell population is mostly comprised of transitional B cells (22). Lymph node B cells are partially depleted by a single dose with the resultant B cells showing switched memory phenotype (108).

Basilixumab, a chimeric IgG1 monoclonal antibody against the α-peptide chain of the IL-2Rα (also referred to as CD25), prevents IL-2-mediated proliferation of T lymphocytes, a critical step in the cellular immune response (18). Interestingly, peripheral B cells express IL-2Rα, and IL-2 signaling is critically involved in plasma cell differentiation (19). Basilixumab induction causes minimal B-cell depletion but increases the proportion of naïve B cells (15).

### Triple Immunosuppression

Triple immunosuppression, consisting of a calcineurin inhibitor (CNI), an antiproliferative, and a glucocorticoid, is the most common maintenance immunosuppression regimen for kidney transplantation, used in over 60% of cases (4).

CNIs, such as tacrolimus and cyclosporine, bind to intracellular protein FKBT 12, thus inhibiting calcineurin, which subsequently prevents the dephosphorylation and activation of nuclear regulator NFAT, resulting in decreased IL2 production and IL2R expression. CNIs are the backbone of many commonly used immunosuppressive regimens, with some studies such as the ELITE-Symphony trial, showing higher rates of allograft survival and lower rates of antibody rejection with tacrolimus over cyclosporine (109). CNIs act directly on T cells and have been found to inhibit ICOS^+^PD1^+^ T follicular helper cell (Tfh) differentiation, thus inhibiting B cell proliferation and differentiation (23–25). Specifically, CNI administration attenuates T cell costimulatory ligand (CD40L and ICOS) expression and production of B cell stimulatory cytokines such as IL21, resulting in decreased B cell activation, plasmablast differentiation, and therefore immunoglobulin secretion in autologous T-B cell co-cultures (24, 26). However, as CNIs do not act directly on B cells, CNI treatment does not inhibit immunoglobulin production from B cells cultured with preactivated T cells (26). Accordingly, preclinical *in vivo* experiments have shown depletion of GC Tfh, decreased expression of IL21, and subsequent prevention of DSA and ABMR with high dose CNI treatment (27).

Mycophenolate mofetil (MMF) and mycophenolic acid (MPA) directly inhibit T- and B-cell proliferation *via* the inhibition of guanine nucleotide synthesis. MMF also modulates B cell activation and differentiation indirectly through the downregulation of T cell protein CD40L and suppression of cytokine production (26, 28). Furthermore, MMF directly inhibits B cell expansion and plasma cell differentiation when applied to primed B cells; immunoglobulin production is also reduced with MMF treatment (28, 31). However, MMF does not suppress immunoglobulin production from terminally differentiated plasma cells (28).

Glucocorticoids, such as prednisone, modulate both the innate and adaptive immune responses and suppress inflammation *via* many mechanisms, including induction of anti-inflammatory genes and inhibition of nuclear regulators such as NF-κB (110). Steroid act both directly on B cells, decreasing antibody production and inducing apoptosis, and indirectly *via* the suppression of dendritic cell and T cell populations (29, 30).

### Rapamycin

Rapamycin, a mammalian target of rapamycin (mTOR) inhibitor, is used as an alternative to CNIs in maintenance immunosuppressive regimens. mTOR inhibitors suppress T cell activation and differentiation *via* blockade of cell-cycle progression (111), inhibition of thymic maturation (112), and downregulation of chemokine signaling (113, 114). Interestingly, rapamycin treatment results in a decrease in Tfh and effector T cells, with a relative increase in CD4^+^CD25^+^FOXP3^+^ regulatory T cells, which are implicated in attenuating memory T cell proliferation (32–34). However, one important advantage of mTOR inhibitors over CNIs is the direct suppression of GC formation, B cell proliferation and immunoglobulin production (31, 35, 36). In hindering GC activation, rapamycin effectively prevents B cell activation and subsequent differentiation to the plasma cell phenotype; however, treatment does not suppress antibody production from already differentiated plasma cells, suggesting that mTOR is necessary for B cell activation and proliferation, but not for memory response (35, 37).

### Costimulation Blockade

Belatacept is a CTLA-4-Ig fusion protein that binds CD80 and CD86 on APCs, thus inhibiting binding to CD28, which suppresses T cell activation and subsequent dependent B cell response (38). Specifically, belatacept decreases ICOS^+^PD1^+^ Tfh populations, resulting in decreased B cell maturation and plasmablast differentiation in *in vitro* experiments (39). Preclinical studies in nonhuman primates also demonstrated suppression of Tfh, and thus GC B cell clonal expansion (40). Transplant recipients treated with belatacept had lower levels of circulating Tfh, memory B cells, and plasmablasts compared to those treated with CNIs (39). In addition to suppressing GC activation by interrupting Tfh-B cell cross talk, experiments in murine models have shown that belatacept treatment disrupts established GCs and reduces alloantibody production even when T cell priming has already occurred (41, 42). Accordingly, belatacept treatment inhibits memory alloreactive B cell and thus recall antibody responses in sensitized recipients (115). Clinically, belatacept has been approved for use in kidney transplantation, with similar rates of acute rejection, graft survival, and death compared to CNIs (116, 117). It has also been used as maintenance monotherapy in immunologically low risk (no DSA, cPRA<20%) patients following alemtuzumab and one-year belatacept/rapamycin dual therapy (118). *Post-hoc* analysis of BENEFIT and BENEFIT-EXT studies revealed that belatacept-treated patients had lower incidence of *de novo* DSA compared to cyclosporine-treated patients (119).

Blockade of the CD40/CD40L costimulatory pathway has been extensively evaluated in kidney transplantation following reports of prolonged graft survival in NHP with an anti-CD40L antibody either in combination with CTLA4-Ig (120) or as monotherapy (121). Translation of anti-CD40L agents was halted following thromboembolic events due to high fragment crystallizable (Fc) effector function activity and binding to platelets (122–125). Since then, several anti-CD40L antibodies without Fc effector function have been engineered with improved safety profiles. The humoral response is controlled through several distinct mechanisms. CD40 is constitutively expressed on B lymphocytes (126), and several anti-CD40 agents lead to B cell depletion through Fc effector function (40, 127). Furthermore, blockade of the CD40/CD40L pathway disrupts GC responses (128), and was shown to prevent early DSA and ABMR in NHP (40). Importantly, Fc-silent anti-CD40 lacking the ability to deplete B cells still promotes long-term graft survival in NHP, underlying the crucial role of GC disruption in achieving favorable clinical outcomes (129). Several clinical trials evaluating agents blocking the CD40/CD40L pathway are either on-going (NCT05027906, NCT04046549) or recently ended (NCT03663335, NCT02217410) and will provide insight into their efficacy in preventing the humoral response in humans.

### Limitation of Current Immunosuppressive Regimens

Belatacept-based maintenance therapy has emerged as an alternative immunosuppressive regimen with a more favorable side effect profile (i.e. less nephrotoxicity and posttransplant malignancies) compared to CNI-based maintenance therapy. Indeed, the BENEFIT trial revealed a 43% reduction in risk of death of graft loss and enhanced renal function at 7 years for belatacept-based regimens compared to CNI-based regimens (130). Nevertheless, the primary limitation of belatacept-based immunosuppression is that it is less effective in preventing early acute cellular rejection compared to CNI-based immunosuppression (131, 132), including when patients previously maintained on CNI-based therapy were switched to belatacept-based therapy (133). Several subsets of mature T cells have been associated with costimulation blockade resistant rejection (CoBRR) such as CD57+PD1-CD4+ T cells (134, 135) and CD28^+^CD4^+^ memory T cells (136, 137). Steroid-sparing, belatacept-based immunosuppression following depletional induction as well as addition of rapamycin to a belatacept-based regimen have reduced rates of acute rejection in small uncontrolled single-center clinical trials, suggesting that a belatacept-based regimen could yield similar rates of acute rejection compared to CNI-regimen if properly optimized (138).

Another limitation is that belatacept is given once monthly intravenously, which may limit compliance and contribute to the generation of *de novo* DSA (6). As a result, belatacept continues to be seldom used in renal transplantation with less than 10% of all adult transplant recipients on maintenance belatacept (4).

Most renal transplant patients in the U.S. are currently managed long-term with tacrolimus, mycophenolate, and often steroids. While effective, especially short-term, this regimen requires drug adherence and that drug levels of tacrolimus be maintained above 5 ng/ml in order to avoid risk of antibody-mediated rejection (139). When tacrolimus dosing level is lowered to less than 5 ng/ml (mostly due to side-effect avoidance, socioeconomic reasons, or non-adherence) ABMR becomes more likely. DSA development in the late post-transplant period, in association with overt or subclinical rejection, is difficult to clear using current therapies, and most of the time results in graft injury. This scenario occurs in 30-40% of patients at some point after their kidney transplant, and better therapeutic options are needed to avoid graft loss. Graft loss implies return to dialysis and need for retransplantation, further stressing the already insufficient supply of donor kidneys. Therefore, a better solution to management of DSA and ABMR is needed to extend the lives and quality of life of at least a third of renal transplant patients, reduce the need for retransplantation, and reduce this source of demand for donor kidneys—now the third largest cause of end-stage renal failure in the U.S. Fortunately, there are several on-going or upcoming clinical trials in the USA that aim at rigorously investigate novel treatments for both desensitization and ABMR (**Table 2**).

**Table 2:** Summary of ongoing clinical trials for ABMR and desensitization treatment.

Trial	Drug	MOA	Application	Primary outcome	Select secondary outcomes	
**IMAGINE**	Clazakiumab	Anti IL-6 mAb	Chronic ABMR treatment	All-cause composite allograft loss return to dialysis allograft nephrectomy re-transplantation eGFR<15 death	Change from baseline to end of treatment DSA and MFI scores Banff lesion grading score Incidence of acute TCMR and ABMR episodes	NCT03744910
**ADAPT**	Carfilzomib Belatacept	Proteosome inhibitor CD80/CD86-CD28 CoB	Desensitization in highly sensitized patients	No subject stopping rule for safety AND Remaining free of all of the following through week 20 post-treatment or until receiving transplant ≥grade 3 infusion reaction ≥grade 3 infection malignancy Proportion of subjects who meet ≥1 of the following at week 20 compared to baseline elimination of one HLA antibody ≥50% reduction in MFI of ≥3 HLA antibodies kidney transplant with a previously incompatible donor without graft loss due to ABMR within first 4 weeks post-transplant	Proportion of subjects transplanted within 1 year of starting treatment who are transplanted with a previously incompatible donor with biopsy-proven ABMR Incidence of ABMR events within 1 year compared to baseline mean number of HLA antibodies eliminated mean percent reduction in MFI	NCT05017545
**ATTAIN**	Daratumumab Belatacept	Anti-CD38 mAb CD80/CD86-CD28 CoB	Desensitization in highly sensitized patients		Proportion of subjects transplanted within 1 year of starting treatment who are transplanted with a previously incompatible donor with biopsy-proven ABMR Incidence of ABMR events within 1 year	NCT04827979
**CTOT42** **(CarBel)**	Carfilzomib Belatacept	Proteosome inhibitor CD80/CD86-CD28 CoB	Biopsy-proven ABMR	To be determined	To be determined	To be determined
**Felzartamab** **(in late ABMR)**	Felzartamab	Anti-CD38 mAb	Late ABMR	Incidence of treatment-emergent adverse events	ABMR categories DSA levels Serum Ig levels Transplant glomerulopathy score Glomerulitis plus peritubular capillaritis sum score C4d score Molecular ABMR score and categories	NCT05021484

## Ongoing and Future Clinical Trials to Address Desensitization and Treatment of ABMR

### IMAGINE Trial

The IMAGINE trial (NCT03744910) is an ongoing phase 3 clinical trial to assess the safety and efficacy of clazakizumab for the treatment of chronic ABMR, aiming to enroll 350 kidney transplant patients. Clazakizumab is a humanized monoclonal antibody targeting IL-6 and is administered subcutaneously monthly. IL-6 blockade quiets inflammation and antibody production which may reduce the injury associated with DSA and ABMR (140). A prior phase 2 RCT evaluating clazakizumab in late AMBR showed promising results with early decrease in DSA, slowed eGFR decline, and alleviation—resolution in some patients—of ABMR following clazakizumab treatment (141). The trial was withdrawn due to safety concerns with 25% patients developing serious infectious events, and 10% developing diverticular disease complications. The aforementioned safety events influenced design of the IMAGINE trial which utilized a reduced monthly clazakizumab dose (25 to 12.5mg/kg dose) and stricter exclusion criteria.

The IMAGINE trial has a primary outcome measure of all-cause composite allograft loss, defined as return to dialysis, allograft nephrectomy, re-transplantation, or eGFR <15, or death from any cause. As of this writing, the trial is in active enrollment and randomizes 1:1 to drug vs. placebo. A monthly subcutaneous injection is an attractive route and frequency of drug administration from a patient perspective, and if effective, this treatment would offer a biologic therapy of greater value than currently available. The trial aims to treat a uniform population of patients with chronic ABMR narrowly defined both clinically and by biopsy criteria. One disadvantage of the study design is the long length of time and large number of centers required to achieve target enrollment. Nevertheless, this elegant trial design uses slope of eGFR at one year as a surrogate endpoint for renal allograft survival, and thus represents an advance with respect to adoption of a surrogate endpoint accepted by the FDA in kidney transplant trials (100, 139, 141).

### ADAPT and ATTAIN Trials

The ADAPT trial (NCT05017545) is a desensitization trial for adult patients who are highly allosensitized (cPRA >99.9%) and on the wait list for renal transplantation. This single-center study of 15 patients is also mirrored by the ATTAIN study (NCT04827979) which has very similar trial design, but differs in the drug combinations being tested to reduce alloantibody levels. The ADAPT study aims to evaluate the combination of a proteosome inhibitor, carfilzomib, and an anti-CD28 costimulation blocker, Belatacept, to lower alloantibody levels. ATTAIN will use an anti-CD38 mononclonal antibody called daratumumab in place of carfilzomib to target plasma cells, but will also use belatacept. The ADAPT study will maintain belatacept for one year in study patients while a shorter course is given in the ATTAIN study. The primary endpoint of both trials is fulfilling a subject stopping rule, or remaining free of all of the following through week 20 post-treatment initiation or until receiving a transplant, whichever occurs earlier: a. grade 3 or higher infusion reaction, b. grade 3 or higher infections, and c. any malignancy. The study site will grade the severity of adverse events experienced by the study subjects according to the criteria set forth in the National Cancer Institute’s Common Terminology Criteria for Adverse Events (CTCAE) Version 5.0 (Published November 27, 2017). The ADAPT study and the ATTAIN study are largely based on non-human primate results evaluating these therapies for their efficacy and safety in lowering antibody levels and permitting rejection-free renal transplantation subsequently (142–144). While the primary aims of both studies are to assess safety, the secondary goals are to assess efficacy in lowering antibody levels, leading to renal transplants being allocated to study patients with a negative crossmatch test, and to assess how the treatments affect plasma cells in blood and bone marrow of the subjects.

### CTOT42, CarBel Trial

The CarBel trial or CTOT42 trial is an NIH-sponsored clinical trial for the treatment of active or chronic active ABMR. This trial, also based largely on the results of pre-clinical work in non-human primates (Schmitz et al., submitted), will evaluate the safety and efficacy of carfilzomib/belatacept dual therapy for the treatment of biopsy-proven ABMR. This multi-center, randomized, prospective clinical trial will compare standard of care treatment consisting of intravenous immune globulin (IVIg) with or without therapeutic plasma exchange (TPE) to combined carfilzomib/belatacept. A total of six doses of carfilzomib will be used along with a year of belatacept, measuring the primary outcome at one year using iBox, a tool developed by the Paris Transplant Group, that has been validated by retrospective data (145). This trial is still in the planning stage with intended initiation in late 2022. The trial includes mechanistic aims to assess the impact of therapy on allo-specific B memory cells and plasma cells derived from blood and bone marrow. Additionally, digital imaging pathology will be used to evaluate artificial intelligence methods to diagnose ABMR and compare such signatures to pathologists’ interpretation of kidney biopsies.

### Felzartamab in Late ABMR

Felzartamab, a recombinant fully human monoclonal anti-CD38 monoclonal antibody, will be evaluated for safety and tolerability in a multi-center 12-month randomized placebo-controlled parallel-group trial (NCT05021484) (146). The study hypothesis is that felzartamab will deplete DSA-producing plasma cells thus reducing alloantibody production. Additionally, CD38 is expressed on natural killer (NK) cells, and interference *via* felzartamab administration may prevent NK cell-triggered tissue injury seen in ABMR. Doberer et al. recently published a case report highlighting the successful reversal of late ABMR using felzartamab, resulting in stabilization of graft function, disappearance of DSA, improved AMBR score on graft histology, and decrease in peripheral and graft NK cells (147).

This trial will enroll 20 kidney transplant recipients with evidence of late ABMR. Participants will be randomized to receive either felzartamab or placebo for 6 months and evaluated for an additional 6 months, including protocol biopsies at 6 months and 1 year. Primary endpoint is assessment of safety and tolerability. Secondary endpoints include pharmacokinetics/pharmacodynamic profiles, serum/urine biomarkers of rejection, biopsy results, and surrogate parameters of allograft dysfunction (eGFR slope and iBOX). While not powered to detect meaningful effect on clinical outcomes of felzartamab compared to placebo, this trial may provide a useful framework for the design of future larger studies.

## Conclusion

Given the negative impact of allosensitization in renal transplantation, in particular B cell sensitization and alloantibody production, the development of immunosuppression targeting B cell and plasma cell responses is much needed. Such therapies need to be evaluated not only for efficacy, but also for their impact on B cell responses to infectious agents and thus protective immunity. As current immunosuppressive regimens impair protective immunity leading to increased risk of infection and malignancy, it follows that additional immunosuppression would further heighten these risks. Therefore, careful trial design and collaboration with infectious disease experts is necessary to develop such therapies. Nevertheless, the field of B cell and plasma cell biology has grown exponentially in recent decades, resulting in a plethora of drugs that target specific immune cell populations and that can down-regulate the B cell response. Further applications of these increasingly targeted drugs offer an opportunity to improve survival and graft function outcomes for transplant recipients as we learn how to adapt these strategies in the clinical setting.

## Author Contributions

All authors listed have made a substantial, direct, and intellectual contribution to the work and approved it for publication.

## Funding

This work was partially supported by the National Institute of Allergy and infectious Diseases of the National Institutes of Health as part of the NHP Transplantation Tolerance Cooperative Study Group under the U19AI131471 (awarded to S.J.K.). Additionally, this work was supported by NIH T32 AI141342-03 (awarded to IJA) and ASTS Presidential Student Mentorship Grant (awarded to IFD).

## Conflict of Interest

SJK is a consultant to Novartis, Visterra, and Hansa Biopharma. AMJ is a consultant to Novartis and Hansa Biopharma. SJK and JK has received research materials/grants from Alexion and MorphoSys.

The remaining authors declare that the research was conducted in the absence of any commercial or financial relationships that could be construed as a potential conflict of interest.

## Publisher’s Note

All claims expressed in this article are solely those of the authors and do not necessarily represent those of their affiliated organizations, or those of the publisher, the editors and the reviewers. Any product that may be evaluated in this article, or claim that may be made by its manufacturer, is not guaranteed or endorsed by the publisher.
